# Prevalence and Antimicrobial Susceptibility Patterns of Bacterial Pathogens Causing Central Line-Associated Bloodstream Infections in Adult Intensive Care Unit Patients at a Tertiary Care Hospital

**DOI:** 10.7759/cureus.113548

**Published:** 2026-07-28

**Authors:** Mohd Areeb, Ramanath Karicheri, Pushpendra Pathak

**Affiliations:** 1 Medical Microbiology, Index Medical College, Hospital & Research Centre, Indore, IND; 2 Microbiology, Index Medical College, Hospital & Research Centre, Indore, IND

**Keywords:** antimicrobial susceptibility, bacterial pathogens, central line-associated bloodstream infection, gram-negative bacteria, hospital-acquired infection, intensive care unit, multidrug resistance

## Abstract

Background: Central line-associated bloodstream infections (CLABSIs) are important device-associated infections among critically ill patients. The causative organisms and their antimicrobial susceptibility patterns vary between institutions. However, institution-specific data regarding bacterial CLABSIs among adult intensive care unit (ICU) patients in central India remain limited. This study evaluated the occurrence, bacterial profile, and antimicrobial susceptibility patterns of CLABSIs among clinically suspected adult ICU patients with central venous catheters (CVCs).

Materials and methods: A prospective observational study was conducted in the adult ICU of Index Medical College Hospital and Research Centre, Indore, India, from January 2024 to December 2025. A total of 245 consecutively enrolled patients aged >18 years with a CVC in place for >48 hours and clinical suspicion of having a bloodstream infection were included. Blood specimens were obtained aseptically from peripheral veins and CVCs. Blood-culture bottles were processed using the BacT/ALERT 3D automated system, and positive cultures were subcultured onto blood agar and MacConkey agar. Bacterial isolates were presumptively identified using standard biochemical tests and further identified to the species level using the VITEK 2 Compact system. Antimicrobial susceptibility testing was performed using the Kirby-Bauer disc-diffusion method, while colistin susceptibility was determined by broth microdilution, and vancomycin minimum inhibitory concentration was determined by VITEK 2. Results were interpreted according to the CLSI 2024.

Results: Among the 245 ICU patients included in the study, 35 (14.29%) were confirmed to have CLABSI, whereas 210 (85.71%) were CLABSI-negative. The overall CLABSI rate was 13.70 episodes per 1,000 catheter-days. Among positive cases, 23 (65.7%) were male and 12 (34.3%) were female. Most cases occurred in patients aged 61-80 years, and the Cardiac Care Unit had the highest positivity rate. Gram-negative bacilli (GNB) accounted for 26 (74.3%) isolates and Gram-positive cocci (GPC) accounted for nine (25.7%). Among GNB, *Klebsiella pneumoniae* comprised eight (22.9%) isolates, followed by *Acinetobacter baumannii *7 (20.0%), *Pseudomonas aeruginosa *5 (14.3%), *Escherichia coli* 5 (14.3%), and *Proteus mirabilis *1 (2.9). Among GPC, *Staphylococcus aureus *accounted for 7 (20.0%) and *Enterococcus faecalis *2 (5.7). GNB isolates showed resistance, including to carbapenems, but susceptibility to colistin. All GPC were susceptible to vancomycin, linezolid, teicoplanin, and rifampicin. Among seven *Staphylococcus aureus* isolates, four (57.1%%) were cefoxitin-resistant and were classified as methicillin-resistant Staphylococcus aureus.

Conclusions: CLABSIs continue to be notable healthcare-associated infections among ICU patients with CVCs, affecting 35 (14.29%) of the 245 patients included in the study. The prevalence of multidrug-resistant Gram-negative bacteria, particularly *Klebsiella pneumoniae* and *Acinetobacter baumannii*, underscores the necessity for ongoing microbiological surveillance, ICU-specific antibiograms, stringent infection control measures, judicious empirical antibiotic therapy, antimicrobial stewardship, and prompt removal of superfluous central lines to mitigate the incidence of CLABSIs. They may support the development of local ICU-specific antibiograms and institutional infection-control and antimicrobial-stewardship policies.

## Introduction

According to CDC-NHSN (Centers for Disease Control and Prevention-National Healthcare Safety Network criteria), central line-associated bloodstream infections (CLABSIs) are defined as laboratory-confirmed bloodstream infections under particular conditions: a observed pathogen is detected in one or more blood cultures after 48 hours following central venous catheter (CVC) insertion, provided the pathogen is not linked to an infection at another site, and common skin commensals, such as coagulase-negative Staphylococci, Bacillus species, Diphtheroids, micrococci, or Propionibacterium species, are isolated from two or more blood cultures obtained on different occasions, associated with at least one of the following clinical manifestations: fever >38°C, chills or rigors, unexplained hypotension, features of sepsis, altered mental status, or clinical deterioration without an evident non-bloodstream source, as assessed by the treating clinician [1].

If an infection is not present or incubating when a patient is admitted to the hospital but becomes apparent at least 48 hours after admission, three days after discharge, or up to 30 days following surgery, then it is considered a hospital-acquired infection. The predominant hospital-acquired bloodstream infection (HA-BSI) is the CLABSI, in which pathogens enter the circulation through a CVC [2]. CLABSIs are prevalent in acute-care settings. CLABSIs are prevalent in intensive care unit (ICU) patients with central lines, accounting for roughly 35% of infections. CVC-associated bacteremia represents approximately 14% of nosocomial infections and constitutes the predominant source of bloodstream infections, with rates of incidence ranging from 1.6 to 44.6 per 1000 catheter days in low-income countries, in contrast to 1.5 per 1000 catheter days in the United States [3-6]. It is exceedingly common among the adult demographic, particularly those with concomitant conditions [7].

CVCs are increasingly employed at hospitals for the treatment of patients with critical illnesses requiring prolonged intravenous medications, fluid administration, drug delivery, blood product intravenous therapy, nutritional support, hemodynamic surveillance, plasmapheresis, and the use of hemodialysis. CVCs present a heightened risk of infection relative to other indwelling catheters [8]. CLABSIs are more common in patients undergoing surgery, with compromised immune systems, experiencing multi-organ dysfunction, requiring mechanical ventilation or renal replacement therapy, and presenting with greater illness severity at ICU admission [9-11]. CLABSIs are infections mediated by biofilms, as catheter pollution can result in the formation of bacterial and fungal biofilms, which are significant contributors to catheter colonization and bloodstream infections [12].

Potential risk factors for CLABSIs mostly stem from noncompliance with aseptic techniques and insertion bundles during CVC placement, as well as subsequent nonadherence to maintenance bundles post-insertion. The potential risk factors include the method of insertion (emergency insertion presents a higher risk than elective insertion due to less compliance with appropriate bundle care) and the insertion site (subclavian/internal jugular/femoral). Subclavian vein CVCs demonstrate reduced rates of CLABSIs relative to internal jugular CVCs, whereas femoral CVCs exhibit the highest CLABSI rates due to their proximity to the female genital tract, duration of use (short-term/long-term catheters), and the purpose of catheterization [12]. Local risk factors encompass insufficient personal hygiene, occlusive transparent dressings, and moisture at the exit site. Nasal colonization by *Staphylococcus aureus* and related illnesses highlight the importance of bacterial colonization in disease development. Supplementary risk factors include advanced age, prior diseases, comorbidities, and recent hospitalization or surgical interventions [13]. The bacterial pathogens responsible for CLABSIs and their antimicrobial susceptibility patterns vary across hospitals and geographical regions. The predominant organisms responsible for CLABSIs are *Klebsiella species, Acinetobacter species, Proteus species, Pseudomonas aeruginosa, Escherichia coli, Staphylococcus aureus, coagulase-negative Staphylococcus, *and *Enterococcus species* [14]. Currently, there is a rising incidence of CVC infections attributed to Gram-negative bacteria [15]. There is a significant prevalence of multidrug resistance (MDR) associated with CLABSIs [16].

Patients with CVCs, aside from CLABSIs, are susceptible to additional infectious problems such as local insertion-site infections, septic thrombophlebitis, and other metastatic infections [13]. The most severe complications of CLABSIs are bacteremia, sepsis, and mortality [17]. It may induce endocarditis in as many as 23% of patients or more [18,19].

The prevention of CLABSIs necessitates the development of guidelines, recommendations, care packages, ideal sterile barriers, the use of two percent chlorhexidine, prompt catheter removal, and the utilization of anti-microbial catheters and anti-microbial catheter lock solutions [20].

The increasing incidence of multidrug-resistant bacteria has hindered therapeutic endeavors. MDR is defined as acquired non-susceptibility to at least one antimicrobial agent in three or more antimicrobial categories. 

Local microbiological surveillance is therefore necessary to describe the organisms circulating within individual healthcare facilities. However, institution-specific data on the prevalence, bacterial profile, and antimicrobial susceptibility patterns of CLABSIs among adult ICU patients in this region remain limited.

This study aimed to determine the prevalence of CLABSIs among adult ICU patients with CVCs, identify the bacterial pathogens isolated, and describe their antimicrobial susceptibility patterns at a tertiary-care hospital.

## Materials and methods

Study design and setting

The prospective observational study was undertaken in the intensive care units of Index Medical College Hospital and Research Centre, Malwanchal University, Indore, Madhya Pradesh, India, from January 2024 to December 2025. The study encompassed ICU patients with CVCs who were clinically suspected of acquiring bloodstream infections during their hospitalization. The Department of Microbiology conducted a microbiological analysis of the gathered samples. During the study period, the sample size was calculated using the single-proportion formula, considering an expected CLABSI prevalence of 15%, a 95% confidence level, and 5% absolute precision. The minimum required sample size was 196. After adjusting for a 20% anticipated dropout or incomplete data, the final sample size was 245 ICU patients with central lines. Two hundred forty-five eligible patients were enrolled consecutively during the study period based on predetermined inclusion criteria, with the sample size determined by prior prevalence estimates and the availability of qualifying cases.

Methodology

Adult patients aged 18 years who were admitted to the ICU during the study period and had a CVC in place for more than 48 hours were included. Eligible patients had developed signs and symptoms suggestive of a HA-BSI after 48 hours of hospital or ICU admission, and their blood cultures were submitted to the microbiology laboratory for suspected CLABSIs. Only patients who provided informed consent to participate were enrolled in the study, and those who exhibited a positive blood culture within 48 hours of hospital admission, presented indications of a community-acquired infection, or had incomplete clinical or microbiological data were excluded from the study.

Sample collection

Aseptically collected blood samples were obtained from ICU patients displaying clinical signs of bloodstream infections 48 hours post central line insertion, suspected of CLABSIs and only the first eligible CLABSI episode per patient was included. Two sets of blood cultures were obtained: one from a peripheral vein and the other from the central line catheter. Approximately eight to ten cc of blood was injected into blood culture bottles and immediately dispatched to the microbiology laboratory.

The recorded data include age, sex, type of intensive care unit, date of hospital admission, date of CVC insertion, date of CVC removal, date of blood sample collection, duration of catheterization, blood culture result, bacterial isolate, and antimicrobial susceptibility pattern.

Microbiological processing

Blood-culture bottles were incubated in the BacT/ALERT 3D automated blood-culture system (bioMérieux Pvt. Ltd., India) at 35-37°C according to the manufacturer’s instructions. Bottles flagged as positive by the instrument were aseptically subcultured onto blood agar and MacConkey agar. The inoculated culture plates were incubated aerobically at 35-37°C for 18-24 hours. Bacterial isolates were presumptively identified on the basis of colony morphology, Gram-staining, and standard biochemical reactions and at the species level by the VITEK 2 Compact automated identification system (bioMérieux Pvt. Ltd., India), with GN identification card used for Gram-negative bacilli isolates, and GP identification card used for Gram-positive cocci. The AST-N405 and AST-N406 card was used for Gram-negative bacilli, whereas the AST-GP628 card was used for Gram-positive cocci (*Staphylococcus and Enterococcus*).

Antimicrobial sensitivity test

Routine antimicrobial susceptibility testing was performed using the Kirby-Bauer disc-diffusion method on Mueller-Hinton agar, and broth microdilution for colistin was also performed separately. Vancomycin sensitivity was determined by the VITEK 2 compact system. Broth microdilution involved incubating bacterial inoculum with serial antimicrobial dilutions to determine MIC, while the VITEK 2 method used a standardized suspension processed automatically for identification and susceptibility per CLSI criteria. For disc-diffusion testing, bacterial suspensions equivalent to a 0.5 McFarland standard were inoculated onto Mueller-Hinton agar plates, and antibiotic discs were applied. After incubation at 35±2°C for 16-18 hours, inhibition zone diameters were measured and interpreted according to CLSI M100, 34th edition, 2024 [21,22]. 

For Enterobacterales isolates, including *Klebsiella pneumoniae, Escherichia coli, and Proteus mirabilis,* antimicrobial susceptibility testing by the Kirby-Bauer disc-diffusion method included the following agents: ampicillin (10 µg), piperacillin-tazobactam (100/10 µg), cefuroxime (30 µg), ceftriaxone (30 µg), and cefepime (30 µg), imipenem (10 µg) and meropenem (10 µg), gentamicin (10 µg) and amikacin (30 µg), ciprofloxacin (5 µg) and levofloxacin (5 µg), trimethoprim-sulfamethoxazole (1.25/23.75 µg), and tetracycline (30 µg).

Colistin susceptibility was determined separately using the broth microdilution method according to CLSI guidelines. Colistin susceptibility for *Proteus mirabilis *and ampicillin susceptibility for K. pneumoniae were not determined as they show intrinsic resistance to these antibiotics.

Non-fermenting Gram-negative bacilli including *Pseudomonas aeruginosa and Acinetobacter baumannii* were tested with agents: piperacillin-tazobactam (100/10 µg), ceftazidime (30 µg), cefepime (30 µg), aztreonam (30 µg), gentamicin (10 µg), ciprofloxacin (5 µg), levofloxacin (5 µg), trimethoprim-sulfamethoxazole (1.25/23.75 µg), imipenem (10 µg) and meropenem (10 µg) and colistin by broth micro dilution.

For *Staphylococcus aureus* isolates, the following agents were used: penicillin (10 units), trimethoprim-sulfamethoxazole (1.25/23.75 µg), tetracycline (30 µg), erythromycin (15 µg), clindamycin (2 µg), linezolid (30 µg), and rifampicin (5 µg). Cefoxitin (30 µg) disc diffusion was used as a surrogate marker for methicillin resistance. Vancomycin, teicoplanin, and daptomycin susceptibility among Gram-positive isolates was determined by automated minimum inhibitory concentration (MIC) using the VITEK 2 compact system.

For *Enterococcus faecalis* isolates, the following agents were used: ampicillin (10 µg), penicillin (10 units), high-level gentamicin (120 µg), high-level streptomycin (300 µg), ciprofloxacin (5 µg), levofloxacin (5 µg), tetracycline (30 µg), linezolid (30 µg), and vancomycin, teicoplanin, and daptomycin by MIC.

Statistical analysis

All collected data were entered into Microsoft Excel and analyzed using IBM SPSS Statistics for Windows, Version 29 (Released 2024; IBM Corp., Armonk, New York, United States) in conjunction with descriptive statistics to get accurate results. Categorical variables were expressed as frequencies and percentages. Pearson’s chi-square test was applied to determine the association between ICU type and CLABSI status. A two-sided p-value of <0.05 was considered statistically significant.

The prevalence was calculated using the following formula:

Prevalence of central line-associated bloodstream infection = (Number of confirmed central line-associated bloodstream infection cases/total number of intensive care unit patients with central venous catheters) × 100

Ethics

The study received clearance from the Institutional Ethics Committee of Index Medical College Hospital and Research Center, affiliated with Malwanchal University, under approval number MU/Research/EC/Ph.D./2023/034. Patient confidentiality was preserved during the study, and informed consent was obtained from participants.

## Results

Demographic characteristics

Among the 245 patients included in the study, 132 (53.9%) were male, and 113 (46.1%) were female. Of the 35 CLABSI-positive patients, 23 (65.7%) were male, and 12 (34.3%) were female. The prevalence of CLABSIs within each sex group was higher among male patients, with 23 of 132 male patients (17.4%) testing positive, compared with 12 of 113 female patients (10.6%). The highest number of CLABSI-positive cases was observed in the 61-80-year age group, and each of the 35 CLABSI-positive patients contributed one bacterial isolate. No polymicrobial CLABSI episode was identified, as shown in Table 1 and Table 2.

**Table 1:** Sex-wise distribution of central line-associated bloodstream infection (CLABSI)

Sex	Total cases	Positive cases	Prevalence within group (%)
Female	113	12	10.6
Male	132	23	17.4

**Table 2:** Age-wise distribution of patients with CLABSI CLABSI: Central line-associated bloodstream infection

Age group (years)	Total cases (n=245)	Positive cases (n=35)	Prevalence within group (%)
≤20	9	2	22.2
21–40	69	8	11.6
41–60	78	8	10.3
61–80	71	15	21.1
>80	18	2	11.1

Prevalence of central line-associated bloodstream infections

This study involved 245 ICU patients with CVCs. Among the 245 patients included in the study, 35 (14.29%) were CLABSI-positive, whereas 210 (85.71%) were CLABSI-negative. A total of 2,554 catheter-days were recorded during the study period. As a result, the CLABSI rate was determined to be 13.70 per 1,000 catheter days as shown in Table 3.

**Table 3:** Prevalence of CLABSI among ICU patients CLABSI: Central line-associated bloodstream infection

CLABSI status	Number of patients	Percentage
CLABSI positive	35	14.29%
CLABSI negative	210	85.71%
Total	245	100%

The frequency of CLABSIs in ICU patients with CVCs was determined to be 35(14.29%). The incidence density was reported as 13.70 CLABSI incidents per 1,000 catheter days.

Distribution according to the intensive care unit type

The maximum number of patients were admitted in the Medical Intensive Care Unit, followed by the Surgical Intensive Care Unit, Coronary Care Unit (CCU), and Respiratory ICU. However, the highest CLABSI positivity rate was observed in CCU, where 12 out of 43 patients (27.91%) were CLABSI positive, as shown in Table 4.

**Table 4:** ICU-wise distribution of CLABSI Pearson’s chi-square test demonstrated a statistically significant association between ICU type and CLABSI status (χ² = 10.70, df = 3, p = 0.013). Therefore, the null hypothesis of no association between ICU type and CLABSI status was rejected. CLABSI: Central line-associated bloodstream infection

ICU type	Total patients	CLABSI positive patients	Positivity rate, %	Pearson’s χ² value	p-value
MICU	131	11	8.40%		
SICU	45	8	17.78%		
CCU	43	12	27.91%		
Respiratory ICU	26	4	15.38%		
Total	245	35	14.29%	10.70	0.013

Distribution of bacterial isolates

Among the 35 CLABSI-positive cases, Gram-negative organisms were predominant. Gram-negative bacilli/coccobacilli accounted for 26 isolates (74.3%), whereas Gram-positive cocci accounted for nine isolates (25.7%). *Klebsiella pneumoniae* was the predominant isolate, followed by *Acinetobacter baumannii*, *Pseudomonas aeruginosa, *and *Escherichia coli,* as shown in Table 5 and Table 6.

**Table 5:** Distribution of Gram-positive and Gram-negative bacterial isolates recovered from CLABSI cases (n = 35) CLABSI: Central line-associated bloodstream infection

Gram-stain category	Number	Percentage
Gram-negative	26	74.3
Gram-positive	9	25.7

**Table 6:** Organism-wise distribution of CLABSI isolates CLABSI: Central line-associated bloodstream infection

Organism isolated	Number	Percentage
Klebsiella pneumoniae	8	22.9
Acinetobacter baumannii	7	20.0
Escherichia coli	5	14.3
Pseudomonas aeruginosa	5	14.3
Staphylococcus aureus	7	20.0
Enterococcus faecalis	2	5.7
Proteus mirabilis	1	2.9
Total	35	100%

Antimicrobial susceptibility profile of Enterobacterales and non-fermenting Gram-negative bacilli isolates

The antimicrobial sensitivity test was conducted separately for Gram-negative bacilli (GNB) and Gram-positive cocci (GPC). Among GNB isolates, high resistance was observed against ampicillin, ceftriaxone, cefuroxime, aztreonam, ceftazidime, cefepime, fluoroquinolones, and carbapenems. Better in-vitro susceptibility among GNB isolates was seen with colistin, as shown in Table 7 and Table 8.

**Table 7:** Antimicrobial susceptibility profile of Enterobacterales isolated from CLABSI cases S: susceptible; IR: intrinsic resistance; CLABSI: central line-associated bloodstream infection.

Antimicrobial agent	K. pneumoniae (n=8) %	E. coli (n=5) %	P. mirabilis (n=1) %	Overall Enterobacterales (n=14) %
Ampicillin	IR	0(0.0%)	0(0.0%)	0(0.0%)
Piperacillin-tazobactam	4(50.0%)	2(40.0%)	0(0.0%)	6(42.9%)
Ceftriaxone	0(0.0%)	0(0.0%)	0 (0.0%)	0(0.0%)
Cefuroxime	0(0.0%)	0(0.0%)	0 (0.0%)	0(0.0%)
Cefepime	2(25.0%)	1(20.0%)	1(100.0%)	4(28.6%)
Gentamicin	6(75.0%)	4(80.0%)	1(100.0%)	11(78.6%)
Amikacin	4(50.0%)	3(60.0%)	0(0.0%)	7(50.0%)
Ciprofloxacin	1(12.5%)	3(60.0%)	1(100.0%)	5(35.7%)
Levofloxacin	2(25.0%)	4(80.0%)	1(100.0%)	7(50.0%)
Trimethoprim-sulfamethoxazole	3(37.5%)	5(100.0%)	0(0.0%)	8(57.1%)
Tetracycline	3(37.5%)	3(60.0%)	IR	6(46.2%)
Imipenem	4(50.0%)	3(60.0%)	1(100.0%)	8(57.1%)
Meropenem	4(50.0%)	3(60.0%)	1(100.0%)	8(57.1%)
Colistin	8(100.0%)	5(100.0%)	IR	13(100.0%)

**Table 8:** Antimicrobial susceptibility profile of non-fermenting Gram-negative bacilli isolated from CLABSI cases NFGNB: non-fermenting Gram-negative bacilli; NA: not applicable; CLABSI: central line-associated bloodstream infection.

Antimicrobial agent	A. baumannii (n = 7) %	P. aeruginosa (n = 5) %	Overall NFGNB (n=12) %
Piperacillin-tazobactam	2(28.6%)	3(60.0%)	5 (41.7%)
Ceftazidime	0(0.0%)	4(80.0%)	4(33.3%)
Cefepime	0(0.0%)	4(80.0%)	4(33.3%)
Aztreonam	NA	4(80.0%)	4(80.0%)
Gentamicin	2 (28.6%)	NT	2(28.6%)
Ciprofloxacin	1 (14.3%)	4(80.0%)	5(41.7%)
Levofloxacin	1(14.3%)	4(80.0%)	5(41.7%)
Trimethoprim-sulfamethoxazole	0(0.0%)	NA	0/7 (0.0%)
Imipenem	0(0.0%)	4(80.0%)	4(33.3%)
Meropenem	1(14.3%)	4(80.0%)	5(41.7%)
Colistin	7(100.0%)	5(100.0%)	12(100.0%)

Antimicrobial susceptibility profile of *Staphylococcus aureus *and *Enterococcus faecalis* isolates

All examined GPC isolates exhibited complete sensitivity to vancomycin, linezolid, teicoplanin, and rifampicin. Significant resistance to penicillin and cefoxitin was noted, indicating the existence of methicillin-resistant *Staphylococcus aureus* among the Gram-positive isolates. The exact methicillin-resistant Staphylococcus aureus (MRSA) count must be calculated only among the seven *Staphylococcus aureus* isolates.

Among seven Staphylococcus* aureus* isolates, four (57.1%%) were cefoxitin-resistant and were classified as MRSA as shown in Table 9 and Table 10.

**Table 9:** Antimicrobial susceptibility profile of Staphylococcus aureus isolates from CLABSI cases MRSA, methicillin-resistant Staphylococcus aureus; MSSA, methicillin-susceptible Staphylococcus aureus; CLABSI, central line-associated bloodstream infection. Values are expressed as the number of susceptible isolates divided by the number tested, with percentages in parentheses.

Antimicrobial agent	MRSA (n = 4) %	MSSA (n = 3) %	Overall S. aureus (n = 7) %
Trimethoprim-sulfamethoxazole	3(75.0%)	1(33.3%)	4(57.1%)
Tetracycline	4(100.0%)	3 (100.0%)	7(100.0%)
Penicillin	0(0.0%)	0 (0.0%)	0(0.0%)
Vancomycin	4(100.0%)	3 (100.0%)	7(100.0%)
Linezolid	4 (100.0%)	3(100.0%)	7(100.0%)
Clindamycin	3(75.0%)	2(66.7%)	5 (71.4%)
Erythromycin	3 (75.0%)	3 (100.0%)	6 (85.7%)
Daptomycin	3 (75.0%)	2 (66.7%)	5 (71.4%)
Rifampicin	4 (100.0%)	3 (100.0%)	7 (100.0%)
Teicoplanin	4(100.0%)	3(100.0%)	7 (100.0%)

**Table 10:** Antimicrobial susceptibility profile of Enterococcus faecalis isolates from CLABSI cases HLG: high-level gentamicin; HLS: high-level streptomycin; CLABSI: central line-associated bloodstream infection.  The high-level gentamicin result should be reported as the absence or presence of high-level aminoglycoside resistance rather than as routine gentamicin susceptibility.

Antimicrobial agent	E. faecalis (n = 2) %
Ampicillin	2(100.0%)
Penicillin	2 (100.0%)
High-level gentamicin (HLG)	2(100.0%)
High-level streptomycin (HLS)	2(100.0%)
Ciprofloxacin	0(0.0%)
Levofloxacin	0(0.0%)
Tetracycline	2(100.0%)
Vancomycin	2 (100.0%)
Teicoplanin	2(100.0%)
Linezolid	2(100.0%)
Daptomycin	2 (100.0%)

## Discussion

Central line-associated bloodstream illness is a major device-related healthcare-associated infection in critically ill patients. Patients in intensive care units are especially vulnerable due to severe underlying conditions, prolonged hospitalizations, multiple invasive procedures, frequent catheter manipulations, exposure to broad-spectrum antibiotics, and weakened immune systems. The Centers for Disease Control & Prevention/National Healthcare Safety Network defines a CLABSI as a laboratory-confirmed bloodstream infection that occurs when a central line has been in place for more than two calendar days on the date of the event or the preceding day, and the infection is not linked to another anatomical site [23].

The current investigation revealed an overall frequency of CLABSIs at 14.29%, with 35 positive cases identified among 245 critical care unit patients possessing central venous catheters. The incidence density was 13.70 episodes per 1000 catheter days, derived from a total of 2554 catheter days. This discovery reveals a significant prevalence of CLABSIs in the research environment. The observed rate exceeds that documented by Maqbool et al., who identified an incidence of 9.3 per 1000 catheter days, and Singh et al., who noted 7.2 per 1000 catheter days [24,25]. The current finding aligns with a recent study conducted in an Indian tertiary care ICU, which documented a CLABSI rate of 13.5 per 1000 central line days [26]. The discrepancies among studies may be ascribed to variations in study populations, case definitions, catheter utilization ratios, infection control protocols, adherence to catheter care bundles, types of ICUs, and antibiotic pressures.

The elevated rate observed in this study may reflect the complex clinical characteristics of ICU patients, prolonged catheterization, frequent catheter access, and possible deficiencies in central line maintenance practices. CLABSIs are deemed mostly avoidable when evidence-based insertion and maintenance protocols are routinely applied. These encompass hand cleanliness, maximal sterile barrier measures during insertion, chlorhexidine skin antisepsis, appropriate catheter site selection, aseptic handling of catheter hubs, daily assessment of line requirement, and prompt removal of unnecessary catheters [27]. The results of this study underscore the necessity for ongoing monitoring and rigorous compliance with central line care protocols in all intensive care units.

In the current investigation, male patients represented a larger share of CLABSI-positive cases, including 23 male patients (65.7%) of the positive cases, whereas 12 female patients were CLABSI-positive (34.3%). The frequency in male patients was 17.4%, whereas in female patients, it was 10.6%. A comparable male predominance has been noted in many hospital-based infection studies, although sex alone may not constitute an independent risk factor.

A notable correlation was identified between the kind of ICU and the status of CLABSIs. The coronary care unit exhibited the greatest positivity rate, with 12 out of 43 patients (27.91%) testing positive, followed by the surgical ICU, respiratory ICU, and medical ICU. The correlation between ICU category and infection status was statistically significant (p = 0.013). This variation may result from disparities in patient severity, catheter usage, emergency catheter placement, nurse-to-patient ratios, frequency of catheter handling, and compliance with infection prevention protocols. The diversity in CLABSI rates across ICUs underscores the necessity of unit-specific surveillance instead of solely depending on aggregate hospital infection rates.

The microbiological profile in this study demonstrated a prevalence of Gram-negative organisms. Gram-negative bacilli comprised 26 (74.3%) of isolates, whereas Gram-positive cocci constituted 9 (25.7%). The predominant bacteria isolated were *Klebsiella pneumoniae* 8 (22.9%), followed by *Acinetobacter baumannii* 7 (20.0%), *Staphylococcus aureus* 7 (20.0%), *Pseudomonas aeruginosa 5 *(14.3%), *Escherichia coli* 5 (14.3%), *Enterococcus faecalis* 2 (5.7%), and *Proteus mirabilis* 1 (2.9%). The preponderance of Gram-negative organisms, including 75% of all isolates cultivated from CLABSIs, aligns with numerous studies conducted in Indian critical care environments, particularly highlighting *Klebsiella pneumoniae* and *Acinetobacter baumannii*. *Pseudomonas aeruginosa* and *Escherichia coli* are commonly connected with device-related bloodstream infections [28].

The prevalence of *Klebsiella pneumoniae* and *Acinetobacter baumannii *is clinically significant due to their association with antibiotic resistance, biofilm formation, extended environmental persistence, and outbreaks in critical care settings. The World Health Organization Bacterial Priority Pathogens List 2024 comprises carbapenem-resistant *Klebsiella pneumoniae *and carbapenem-resistant *Acinetobacter baumannii* among high-priority resistant Gram-negative bacteria for immediate public health intervention [29]. The significant prevalence of these organisms in the current study indicates that antibiotic resistance and environmental contamination may significantly contribute to the persistence of CLABSIs in critical care environments.

The antimicrobial susceptibility profile of Gram-negative isolates exhibited significant resistance to frequently utilized antibiotics. Resistance was notably elevated against ampicillin, ceftriaxone, cefuroxime, aztreonam, ceftazidime, cefepime, fluoroquinolones, and carbapenems. The current investigation revealed complete resistance to ampicillin, ceftriaxone, and cefuroxime in the examined Gram-negative isolates. Resistance to aztreonam and ceftazidime was observed in 20 of 26 isolates (76.9%), whereas 16 of 26 isolates (61.5%) were resistant to cefepime. Carbapenem resistance was also high, with 13 of 26 isolates (50.0%) resistant to both meropenem and imipenem. These findings demonstrate a significant prevalence of multidrug-resistant Gram-negative bacteria.

The elevated resistance to third-generation cephalosporins and carbapenems aligns with national antimicrobial resistance patterns. The antimicrobial resistance surveillance data from the Indian Council of Medical Research indicate diminished susceptibility of *Klebsiella pneumoniae* to piperacillin-tazobactam and carbapenems, alongside a progressive decline in susceptibility to imipenem and meropenem over time [30]. The resistance pattern identified in this study restricts the efficacy of numerous commonly employed empirical antibiotics and underscores the necessity for local antibiogram-guided empirical therapy.

The International Society for Infectious Diseases highlights that CLABSI rates persistently remain elevated in resource-constrained environments, correlating with prolonged hospitalizations, greater expenses, and heightened fatality rates [31].

Among Gram-negative isolates, there was complete in-vitro susceptibility to colistin (100%). Although resistance to several antimicrobial agents, including carbapenems, was observed among the Gram-negative isolates, some isolates remained susceptible to carbapenems, aminoglycosides, and piperacillin-tazobactam, indicating that these antibiotics remain valid treatment options for selected multidrug-resistant Gram-negative infections. Their use should be guided by organism-specific susceptibility results, local antibiogram data, infection severity, and antimicrobial stewardship principles to ensure effective therapy while minimizing the risk of further resistance development.

*Staphylococcus aureus* was the dominating bacterium among Gram-positive isolates. Among the seven Staphylococcus aureus isolates, four isolates (57.1%) were identified as MRSA, while three isolates (42.9%) were methicillin-susceptible Staphylococcus aureus. All examined Gram-positive isolates exhibited susceptibility to vancomycin, linezolid, teicoplanin, and rifampicin. This discovery suggests that glycopeptides and oxazolidinones continue to be viable therapeutic alternatives for Gram-positive CLABSIs in the current context. 

The results of this investigation have significant therapeutic and infection control implications. The prevalence of multidrug-resistant Gram-negative pathogens indicates that empirical treatment for suspected CLABSIs should rely on local microbiological data rather than standardized national or international guidelines. Empirical antibiotic protocols must account for ICU-specific pathogen prevalence, previous antibiotic administration, catheter duration, sepsis severity, and risk factors for multidrug-resistant pathogens. Upon obtaining culture and susceptibility results, antimicrobial de-escalation should be implemented to mitigate selection pressure and maintain the effectiveness of last-resort antibiotics.

This study further underscores the significance of preventive interventions. Consistent training of healthcare personnel, rigorous hand hygiene, aseptic catheter insertion, adherence to maintenance bundles, daily evaluation of catheter necessity, and occasional audits with feedback are crucial for diminishing CLABSI rates. 

Limitations

This study has several limitations. It was conducted at a single tertiary care centre, limiting the generalizability of the findings. The number of CLABSI-positive cases and organism-specific isolates was small; therefore, antimicrobial susceptibility results should be interpreted as descriptive local surveillance data. The study included only bacterial pathogens and did not evaluate fungal isolates. Furthermore, only ICU patients with central lines who were clinically suspected of bloodstream infection were enrolled, which may have introduced selection bias and may not reflect the true CLABSI burden among all eligible patients. Future multicentre studies should include comprehensive surveillance of all patients with central lines and larger organism-specific susceptibility analyses.

## Conclusions

CLABSIs remain significant healthcare-associated infections among ICU patients with CVCs. In this study, CLABSIs occurred in a proportion of patients, with Gram-negative organisms, particularly Klebsiella pneumoniae and Acinetobacter baumannii, predominating and demonstrating considerable resistance to several commonly used antimicrobial agents. These findings underscore the importance of continuous microbiological surveillance, stringent infection-prevention practices, timely removal of unnecessary central lines, and antimicrobial stewardship guided by local susceptibility patterns. However, results should be interpreted as local data and confirmed through larger multicenter studies.
